# Predicting Alzheimer's disease CSF core biomarkers: a multimodal Machine Learning approach

**DOI:** 10.3389/fnagi.2024.1369545

**Published:** 2024-06-26

**Authors:** Anna Michela Gaeta, María Quijada-López, Ferran Barbé, Rafaela Vaca, Montse Pujol, Olga Minguez, Manuel Sánchez-de-la-Torre, Arrate Muñoz-Barrutia, Gerard Piñol-Ripoll

**Affiliations:** ^1^Servicio de Neumología, Hospital Universitario Severo Ochoa, Leganés, Spain; ^2^Departamento de Bioingeniería, Universidad Carlos III de Madrid, Leganés, Spain; ^3^Group of Translational Research in Respiratory Medicine, Hospital Universitari Arnau de Vilanova and Santa Maria, Institut de Recerca Biomedica de Lleida (IRBLleida), Lleida, Spain; ^4^Centro de Investigación Biomédica en Red de Enfermedades Respiratorias (CIBERES), Madrid, Spain; ^5^Unitat Trastorns Cognitius, Clinical Neuroscience Research, Institut de Recerca Biomedica de Lleida (IRBLleida), Hospital Universitari Santa Maria, Lleida, Spain; ^6^Group of Precision Medicine in Chronic Diseases, Hospital Nacional de Parapléjicos, IDISCAM, Department of Nursing, Physiotherapy and Occupational Therapy, Faculty of Physiotherapy and Nursing, University of Castilla-La Mancha, Toledo, Spain; ^7^Departamento de Bioingegneria, Instituto de Investigación Sanitaria Gregorio Marañón, Madrid, Spain

**Keywords:** Alzheimer's disease, neurodegeneration, biomechanism, diagnosis, therapeutic target, quantitative polysomnographic signal analysis, CSF biomarkers, Machine Learning

## Abstract

**Introduction:**

Alzheimer's disease (AD) is a progressive neurodegenerative disorder. Current core cerebrospinal fluid (CSF) AD biomarkers, widely employed for diagnosis, require a lumbar puncture to be performed, making them impractical as screening tools. Considering the role of sleep disturbances in AD, recent research suggests quantitative sleep electroencephalography features as potential non-invasive biomarkers of AD pathology. However, quantitative analysis of comprehensive polysomnography (PSG) signals remains relatively understudied. PSG is a non-invasive test enabling qualitative and quantitative analysis of a wide range of parameters, offering additional insights alongside other biomarkers. Machine Learning (ML) gained interest for its ability to discern intricate patterns within complex datasets, offering promise in AD neuropathology detection. Therefore, this study aims to evaluate the effectiveness of a multimodal ML approach in predicting core AD CSF biomarkers.

**Methods:**

Mild-moderate AD patients were prospectively recruited for PSG, followed by testing of CSF and blood samples for biomarkers. PSG signals underwent preprocessing to extract non-linear, time domain and frequency domain statistics quantitative features. Multiple ML algorithms were trained using four subsets of input features: clinical variables (CLINVAR), conventional PSG parameters (SLEEPVAR), quantitative PSG signal features (PSGVAR) and a combination of all subsets (ALL). Cross-validation techniques were employed to evaluate model performance and ensure generalizability. Regression models were developed to determine the most effective variable combinations for explaining variance in the biomarkers.

**Results:**

On 49 subjects, Gradient Boosting Regressors achieved the best results in estimating biomarkers levels, using different loss functions for each biomarker: least absolute deviation (LAD) for the A*β*42, least squares (LS) for *p*-tau and Huber for *t*-tau. The ALL subset demonstrated the lowest training errors for all three biomarkers, albeit with varying test performance. Specifically, the SLEEPVAR subset yielded the best test performance in predicting A*β*42, while the ALL subset most accurately predicted *p*-tau and *t*-tau due to the lowest test errors.

**Conclusions:**

Multimodal ML can help predict the outcome of CSF biomarkers in early AD by utilizing non-invasive and economically feasible variables. The integration of computational models into medical practice offers a promising tool for the screening of patients at risk of AD, potentially guiding clinical decisions.

## 1 Introduction

Alzheimer's disease (AD) is a neurodegenerative disorder that severely affects cognitive function (Masters et al., 2015). Current statistics on AD paint a concerning picture, with epidemiological forecasts suggesting that by 2060, one in three individuals over the age of 85 in the United States will be affected by the disease (Rajan et al., 2021). AD typically begins to develop in the elderly population a decade or more before clinical diagnosis (Villemagne et al., 2013). This asymptomatic stage is followed by a prodromal phase—or Mild Cognitive impairment (MCI) due to AD—characterized by preserved baseline functionality despite cognitive decline, which precedes the onset of established dementia (Masters et al., 2015). Traditionally, a definitive diagnosis of AD is established post-mortem via histological examination, revealing the hallmark neuropathological features of the disease, such as extracellular amyloid plaques and neurofibrillary tangles (Masters et al., 2015; Jack et al., 2018). Remarkably, emerging evidence strongly correlates CSF amyloid*β* (A*β*42), phospho-tau (p-tau), total-tau (t-tau) levels and AD neuropathological lesions, establishing CSF biomarkers as critical for early AD detection with high diagnostic accuracy (Masters et al., 2015; Jack et al., 2018). It is known that AD progresses through multiple stages characterized by neuropathological advancements throughout the brain, with amyloid pathology preceding tau pathology, followed by neurodegeneration (Jack et al., 2018). In this context, the National Institute on Aging and Alzheimer's Association (NIA-AA) guidelines introduced in the year 2018 the AT(N) criteria based on core fluid and neuroimaging biomarkers (Jack et al., 2018), thus redefining the concept of the “Alzheimer's continuum” to represent the biological as well as the clinical progression of the disease. This paradigm shift arises from evidence indicating that the AD continuum encompasses a prolonged asymptomatic phase characterized by sequential alterations in biomarkers (Aisen et al., 2017; Jack et al., 2018). Biomarkers are grouped into three categories based on the pathologic processes each one measures, encompassing amyloid deposition (A), tau aggregates (T), and neurodegeneration (N), with CSF A*β*42 and amyloid PET for amyloid pathology (A); CSF p-tau and tau-PET of tau pathology (T); atrophy in MRI, hypometabolism in FDG-PET, and CSF t-tau for neurodegeneration (N; Jack et al., 2018). The AT(N) framework, relying solely on CSF biomarkers, has shown 85–90% sensitivity and specificity for AD, with combined assessments yielding greater diagnostic accuracy than individual CSF markers (Grøntvedt et al., 2020). Moreover, this approach is more cost-effective compared to imaging biomarkers (Contador et al., 2023).

While neurodegeneration in AD is irreversible and lacks definitive treatment (Conti Filho et al., 2023), early diagnosis can enhance prognosis and improve disease management (Crous-Bou et al., 2017). Consequently, primary intervention strategies now focus on modifiable risk factors such as cardiovascular health and lifestyle choices (Crous-Bou et al., 2017). Notably, recent research has highlighted a history of disrupted sleep, reported years before the onset of cognitive impairment, as a potentially modifiable risk factor for AD (Macedo et al., 2017). Sleep disturbances are connected to cognitive dysfunction and AD pathogenesis, including the accumulation of amyloid proteins and tau phosphorylation (Ahmadian et al., 2018). Chronic partial sleep restriction in rodents has demonstrated an increase in A*β* deposition. The mechanism behind this phenomenon may involve reduced interstitial fluid volume during sleep deprivation, potentially hindering A*β* clearance (Insel et al., 2021). Additionally, acute sleep deprivation in humans has been shown to elevate overnight CSF A*β* levels by 25–30% compared to sleeping controls (Lucey et al., 2018). Loss of slow-wave sleep (SWS) due to partial sleep deprivation has been associated with an acute increase in next-morning CSF A*β* levels (Ju et al., 2017). It is believed that SWS plays a critical role in A*β* turnover, potentially due to increased glymphatic system activity in the brain during this stage (Mander et al., 2015). On the other hand, concerning tau pathology, cerebrospinal fluid tau levels increased by over 50% following sleep deprivation (Holth et al., 2019). Furthermore, chronic sleep deficiency over 2 months has been linked to a more than 50% increase in insoluble tau in the brains of AD patients (Nunomura et al., 2001). These findings suggest that the sleep-wake cycle plays a crucial role in regulating tau levels in the brain, with sleep deprivation contributing to elevated cerebral tau and its pathological spread (Holth et al., 2019). Moreover, a meta-analysis has determined that patients with Obstructive Sleep Apnea (OSA) have double the risk of cognitive decline and/or AD compared to individuals without OSA (Bubu et al., 2017). Additionally, multiple studies support the hypothesis that the relationship between these conditions could be bidirectional and may manifest before the clinical signs of AD become apparent (Yaffe et al., 2011). Thus, identifying markers for sleep disturbances in early AD stages could pave the way for developing preventive strategies targeting neurodegeneration through sleep intervention.

Therefore, the challenge of diagnosing AD in its initial phases is significant, necessitating the discovery of early biomarkers. Recent studies affirm that core CSF biomarkers can identify preclinical AD in asymptomatic individuals (Sabbagh and DeCourt, 2023), predict progression to MCI, and distinguish AD from other dementias (Hampel et al., 2004; De Leon et al., 2006). This underscores their potential utility in clinical settings for diagnosing preclinical AD, although currently core CSF AD biomarkers are not widely utilized in clinical practice for these purposes (Sabbagh and DeCourt, 2023). Nevertheless, CSF collection requires a lumbar puncture, an invasive procedure with contraindications for some individuals, such as those on anticoagulant therapy (Kim, 2022). Additionally, determining CSF biomarkers necessitates high-cost enzyme-linked immunosorbent assay (ELISA) methods using specific antibodies (Dakterzada et al., 2021). Thus, despite their diagnostic value, these techniques may limit the feasibility of widespread screening, highlighting the need for less invasive diagnostic methods.

In this regard, past studies have proposed that electroencephalography (EEG) metrics may serve as predictive, non-invasive biomarkers of AD (Babiloni et al., 2010). These studies typically focus on analyzing awake resting-state EEG signals for spectral content, complexity, and synchronization (Gallego-Jutglá et al., 2015). Notably, certain EEG spectral power profiles have been associated with traditional CSF biomarkers in the early stages of AD (Chino-Vilca et al., 2022). Researchers have increasingly shown interest in analyzing sleep EEG. EEG changes associated with AD during sleep, such as REM sleep slowing, reduced spindle and K-complex amplitude and duration and disrupted slow-wave activity (SWA) during NREM sleep are well-documented (Petit et al., 2004). Some of these alterations were intriguingly linked to tau and amyloid protein accumulation (Winer et al., 2019).

In recent years, advances in computational neuroscience have been applied to the sleep EEG signal analysis in AD, based on quantitative EEG (qEEG) feature extraction using diverse algorithms (Geng et al., 2022; Azami et al., 2023). Notably, a seminal study demonstrated that specific qEEG measures during sleep could distinguish AD dementia patients from those with MCI and healthy controls (HC), identifying these measures and potential electrophysiological biomarkers of AD (Azami et al., 2023). However, despite the acknowledged impact of sleep on AD neuropathology (Yaffe et al., 2011; Ahmadian et al., 2018), few studies extend these methods to polysomnography (PSG; Khosroazad et al., 2023). PSG is a non-invasive examination that allows for both qualitative and quantitative assessment of various parameters (Berry et al., 2015), thus potentially offering additional insights alongside other biomarkers. Given the complex nature of AD, it is becoming increasingly clear that relying on a single type of biomarker may not suffice for accurate diagnosis.

Consequently, a multimodal approach that incorporates various categories of data is needed and Machine Learning (ML) technologies could address these challenges (Borhani et al., 2022). ML is a subset of artificial intelligence, employing algorithms to parse data and uncover underlying insights. Unlike classical statistical methods, where the focus is on predetermined data patterns, ML enables computational models to identify and learn from data patterns that may otherwise go unnoticed (Bzdok et al., 2018). Additionally, recent advancements in artificial intelligence have made the analysis of large, multimodal datasets not only more efficient but also more clinically relevant (Obermeyer and Emanuel, 2016). Therefore, ML models hold promise in sleep medicine, where vast amounts of electrophysiological data are generated during PSG recordings (Obermeyer and Emanuel, 2016; Khosroazad et al., 2023). This innovative approach has been applied in AD research for diagnosis, progression prediction or neurodegeneration detection, integrating various potential biomarkers such as clinical and neuroimaging data, neuropsychological test results and rest EEG spectral features (Bandyopadhyay and Goldstein, 2023). Nevertheless, models that incorporate sleep-related variables to detect AD neuropathology at an early stage of the disease have not yet been fully explored.

Based on these considerations, we hypothesize that ML techniques could enable us to identify, within a mild-moderate AD population, a specific array of diverse non-invasive variables—including those related to sleep—as potential indicators of AD neuropathology. Our research is thus directed at developing a multimodal ML model to serve as an early diagnostic tool that can accurately and non-invasively predict levels of CSF AD core biomarkers. This is critical for identifying AD-specific neuropathological changes and providing novel insights for a non-invasive clinical protocol to monitor AD-related neuropathology. To this end, we will train various ML models incorporating different subsets of non-invasive variables. These include conventional PSG parameters, quantitative PSG signal features and a range of clinical variables related to AD pathogenesis, such as comorbidities, sociodemographic, and sleep-related information.

## 2 Materials and methods

**Figure 1:**
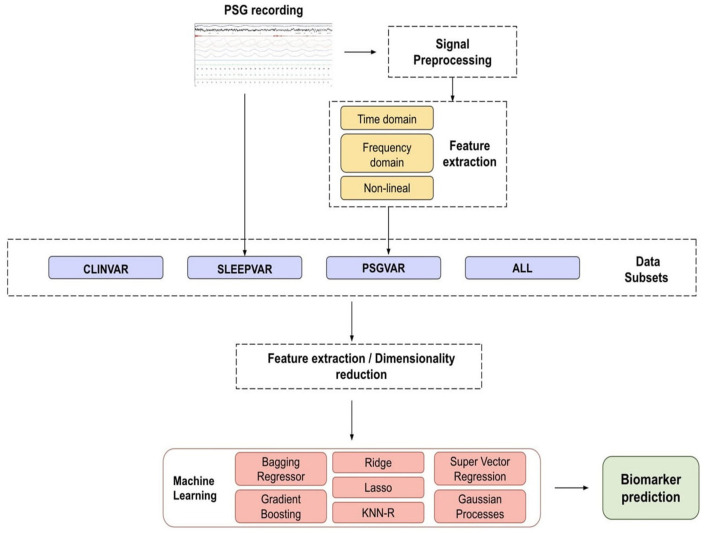
Flowchart of the proposed methodology for biomarker prediction. Our biomarkers are the concentration of A*β*42, t-tau, and p-tau proteins present in the cerebrospinal fluid. The main steps of the proposed methodology are signal processing, feature extraction, dimensionality reduction, and the prediction of biomarker levels using Machine Learning (ML) models. ALL, all the subsets combined; CLINVAR, clinical variables; KNN-R, k-nearest neighbors; SLEEPVAR, conventional PSG parameters; PSG, polysomnography; PSGVAR, quantitative PSG signal measures.

Figure 1 presents a flowchart outlining the study methodology and the ML analysis process for predicting CSF biomarkers.

### 2.1 Study population

We performed an additional analysis based on the longitudinal study NCT02814045, which monitored cognitive progression in a cohort with mild-moderate AD. The study adhered to the principles of the Declaration of Helsinki and received approval from the ethics committee of Hospital Arnau de Vilanova de Lleida (CE-1218). From 2014 to 2018, the Cognitive Disorders Unit at Hospital Universitari Santa Maria (Lleida, Spain) systematically enrolled patients who were over 60 years old, drug naïve and diagnosed with mild-moderate AD. The diagnosis of AD was formulated according to clinical criteria established by the National Institute of Aging Alzheimer's Association (NIA-AA) guidelines (McKhann et al., 2011). We employed the Mini-Mental State Examination (MMSE) tests to assess the severity of cognitive impairment. Exclusively patients exhibiting MMSE ≥ 20 were included (Folstein et al., 1975; Perneczky et al., 2006; Lanctôt et al., 2024). Consent for participation was obtained from the patients themselves, their responsible caregivers, or legal guardians. Exclusion criteria were set to omit individuals with any condition that could interfere with adherence to the study protocols. These exclusion criteria included the presence of sleep disorders, unexplained daytime sleepiness; severe organic or psychiatric conditions, alcohol consumption exceeding 280 g/week; significant central nervous system disease other than AD; untreated deficiencies in vitamin B12 or folate, and untreated thyroid disease. Furthermore, patients who had taken medications such as neuroleptics, hypnotics, antidepressants, or beta-blockers within 15 days before actigraphy were also excluded from the study. Eligible patients underwent thorough overnight PSG assessments. Subsequently, CSF and blood samples were collected the following morning to evaluate biomarker levels. We gathered anthropometric and clinical data including age, gender, body mass index, cardiovascular risk factors, years of education, and toxic habits. Daytime sleepiness was measured using the Epworth sleepiness Scale (ESS), with a score greater than 10 indicating excessive drowsiness (Bzdok et al., 2018). Supplementary Table 1 provides a detailed listing of the sociodemographic, anthropometric, and clinical characteristics collected.

### 2.2 Cerebrospinal fluid biomarkers and ApoE genotyping

CSF samples obtained via lumbar puncture and amounting to 8–10 mL, were centrifuged at low speed (2,000 x *g* for 10 min at 4°C) to pellet any cellular elements. They were then aliquoted in polypropylene tubes before being frozen and stored at −80°C. The samples were then processed for biomarker analysis. Concentrations of CSF t-tau, threonine 181 p-tau, and A*β*42 were determined using enzyme-linked immunosorbent assays (Innotest^®^, Fujirebio, Belgium), as directed by the manufacturer. All samples were tested in duplicate, and the results were given in parts per million (pg/ml). IRBLleida Biobank (B.0000682) and Plataforma Biobancos PT17/0015/0027 facilitated the sample collection process. The A*β*42 cut-off values were 600 pg/ml, with values below this suggesting amyloid deposition. The cut-off values for t-tau and p-tau were set at 425 and 65 pg/ml, respectively. Values exceeding these cut-offs indicated the presence of neurofibrillary tangles. Concurrent with the CSF collection, blood samples were drawn, processed immediately for analysis, and examined.

The ApoE genotype was determined using a Maxwell^®^ RCS blood DNA kit (Promega, USA) and 20 μL of DNA from a 2 mL peripheral blood sample. Participants were categorized as either homozygous or heterozygous carriers of the ApoE4 allele (noted as ApoE4+), with phenotypes 2/4, 3/4, or non-carriers (noted as ApoE4-), with phenotypes 2/2, 2/3, and 3/3.

### 2.3 Polysomnography

Overnight supervised PSG recordings were performed using Philips Respironics Alice 6 equipment to determine sleep parameters. The methodology for sleep recording, technical specifications, and manual grading of raw data was established in accordance with published standards (Berry et al., 2015). The PSG study included the simultaneous recordings from the following electrophysiologic channels: six electroencephalograms (EEG) leads (F3-A2/F4-A1; C3-A2/C4-A1; O2-A1/O1-A2), two bilateral electrooculographic leads (EOG), a single chin electromyogram (EMG) channel, sensors for chest and abdominal respiratory effort, airflow measurements (obtained through both an oral and nasal thermocouple and nasal pressure records), pulse oximetry, an electrocardiogram (ECG), body position sensor, a snoring detection microphone and bilateral piezoelectric sensors detect leg movement detection. Experts manually scored the raw data, extracting sleep and respiratory parameters in line with the established literature (Berry et al., 2015). The physicians' annotations included details on any artifacts observed during the recordings. Measures of sleep quality were determined and included total sleep time (TST), sleep efficiency (SE)—calculated as the percentage of TST relative to the time spent in bed (TIB)—and arousal index (AI), defined as the total number of arousals per hour of sleep. Apneas were identified using an oronasal thermal sensor and defined as a reduction in the airflow sensor signal by 90% when compared to the pre-event baseline value for more than 10 s. Hypopneas were recognized as a decrease of at least 30% in airflow, as measured by nasal pressure, persisting for over 10 s, and associated with either arousal or a 3% decrease in oxygen saturation from the pre-event baseline. The apnea–hypopnea index (AHI) was defined as the average number of apnea and hypopnea episodes per hour of TST. The oxygen desaturation index (ODI) is the average number of instances per hour of sleep where oxygen saturation decreases by 3% or more. The time with oxygen saturation below 90%, referred to as CT90%, is expressed as a percentage of the TST. An AHI exceeding 15 events per hour is indicative of obstructive sleep apnea (OSA).

### 2.4 Signal preprocessing

The MATLAB^®^ signal processing toolbox was used to preprocess and analyze signal data offline. To standardize the format of the signals across different PSG channels and to mitigate the effects of the noise during the analysis phase, an initial preprocessing was conducted. The preprocessing phase was divided into three main steps:

- Resampling: due to varying sample rates across the channels and subjects, signals were resampled for uniformity based on international guidelines (Bandyopadhyay and Goldstein, 2023), as is detailed in Table 1. Subsequently, each signal was decomposed into five new signals corresponding to the sleep phases: W, N1, N2, N3, and REM phases. Segments identified as “Wake” were discarded. The remaining segments, identified as the same sleep stage in their respective hypnogram, were concatenated to form the new signals. These were then resampled at a standard rate of 128 Hz to diminish their size. The resampled signals were further divided into uniform 10-s segments.
- Filtering: the channels were filtered with a fifth-order bandpass digital Butterworth filter (Butterworth, 1930). The cutoff frequencies for each channel were set in accordance with the AASM guidelines (Berry et al., 2015), as specified in Table 1, to ensure comparably meaningful spectral content across channels.
- Artifact removal: based on the annotations provided by expert physicians, segments containing artifacts were identified and excluded from the signals.

**Table 1:** Signal frequency ranges and filter settings: the original sample rate (fs0), the target sample rate (fst), the output sample rate (fsf), and the filter frequencies for the Electrooculogram (EOG), Electroencephalogram (EEG), Electromyogram (EMG), Respiratory effort (Effort), Airflow, Pulse, Oxygen saturation (SpO_2_), Electrocardiogram (ECG), and Snore channels.

**Signal**	**fs0 (Hz)**	**fst (Hz)**	**fsf (Hz)**	**Filter (Hz)**
EOG	200–500	500	500	0.3–50
EEG	200–500	500	500	0.3–50
EMG	200–500	500	200	1–90
Effort	100	100	100	-
Airflow	100	100	100	0.1–15
Pulse	1	1	1	-
SpO_2_	1	1	1	-
ECG	200–500	500	200	0.3–50
Snore	500	500	500	1–100

### 2.5 Feature extraction

Utilizing various computational methods (Álvarez et al., 2012; Alvarez et al., 2013; Umut, 2016), the preprocessed PSG signals were parameterized with features from linear (time-domain and frequency domain) and non-linear features. Each feature set provides unique insights into the signals' characteristics. Time-domain statistics offer information on the signal amplitude, while frequency-domain statistics leverage the spectral information within the signals (Dressler et al., 2004). In contrast, non-linear features shed light on the temporal complexity and regularity of the signals (Abásolo et al., 2006; Furui et al., 2020). Features were calculated as the mean across each sleep stage (N1, N2, N3, and REM) for the entire duration of the overnight recording. For segments annotated as distinct sleep stages, parameters were derived from the mean values within 2-min windows, employing a 50% overlap strategy.

For the time-domain analysis of all PSG signals, we calculated four time domain and four non-linear parameters. Time domain features included Root Mean Square, variance, skewness, kurtosis, and maximum value (Álvarez et al., 2012; Alvarez et al., 2013; Gerla et al., 2019). The non-linear parameters included Shannon Entropy (ShanEnt), Sample Entropy (SampEnt), Lempel Ziv (LempZiv), and Higuchi Fractal Dimension (HFD; Alvarez et al., 2006, 2013). Frequency-domain statistics and spectral parameters (Dressler et al., 2004; Alvarez et al., 2006; Pedregosa et al., 2011) were explicitly calculated for the EOG, EEG, EMG, Airflow, and SpO_2_ channels (see Table 2). The spectral parameters were derived from the Power Spectral Density (PSD) of each normalized 2-min segment (Gerla et al., 2019). The estimates were obtained using Welch's averaged, modified periodogram method with a Hamming window of 65,536 points for EOG, 32,768 for EMG signals, 16,384 for the airflow signals, and 128 for the SpO_2_ signal.

**Table 2:** Frequency domain statistics and spectral parameters computed for Electrooculogram (EOG), Electroencephalogram (EEG), Electromyogram (EMG), and the Airflow and Oxygen saturation (SpO_2_) channels of the polysomnography.

**Signal**	**Components**
EOG	MF 0.5–30 Hz, DF 0.5–30 Hz, Pw 0.5–30 Hz (TotalPw)
	EEG	MF 0.5–30 Hz, DF 0.5–30 Hz, Pw 0.5–30 Hz (TotalPw),
	Pw 0.5–4 Hz (delta), Pw 4–7 Hz (theta), Pw 8–12 Hz (alpha),
	Pw 14–22 Hz (lowBeta), Pw 23–30 Hz (highBeta),
Pw 14–30 Hz (beta), Pw 31–40 Hz (gamma), Pw 12–14 Hz (spindles)
EMG	MF 0.5–30 Hz, DF 0.5–30 Hz, Pw 0.5–30 Hz (TotalPw)
Airflow	MF 0.025–0.05 Hz, DF 0.025–0.05 Hz, Pw 0.025–0.05 Hz (TotalPw)
SpO_2_	MF 0.014–0.033 Hz, DF 0.014–0.033 Hz, Pw 0.014–0.033 Hz (TotalPw)

MF, mean frequency; DF, Dominant frequency; Pw, Power.

## 3 Statistical analysis

Statistical analyzes were conducted using R statistical software, version 3.3.1. Descriptive statistics were calculated for both normally and non-normally distributed quantitative data, with the former presented as mean and standard deviation (SD), and the latter as median with interquartile range (IQR). For categorical variables, we reported absolute and relative frequencies. In the case of quantitative variables, we computed Pearson's correlation coefficient to assess the relationship with the target biomarker. For categorical features, a one-way ANOVA test was employed to determine if there were significant differences between the means of two or more groups. To eliminate irrelevant, correlated, and noisy data, a feature selection process was undertaken. This step aimed to reduce the dataset dimensionality with minimal loss of significant information. We performed feature selection on the training partition of the dataset, organizing the variables into four subsets as follows:

- CLINVAR: sociodemographic, anthropometric, and clinical variables.
- SLEEPVAR: conventional PSG parameters.
- PSGVAR: quantitative measure derived from PSG signals.
- ALL: a combination of all the above subsets.

For the CLINVAR, SLEEPVAR, and PSGVAR subsets, any feature that was missing in more than 50% of the samples was excluded. An initial feature selection was conducted by retaining only those features that exhibited a correlation coefficient with the target biomarker about 0.1 for CLINVAR and SLEEPVAR and above 0.3 for PSGVAR. The ALL subset was then refined to include only features meeting these criteria. Subsequently, we calculated the correlation coefficient for each pair of features, opting to retain only one variable from pairs where the correlation exceeded 0.9, to avoid redundancy. Further dimensionality reduction was accomplished through principal component analysis (PCA). In this process, we chose to retain enough components to explain 90% of the variance, creating new subsets for CLINVAR, SLEEPVAR, PSGVAR, and ALL based on the selected principal components. Additionally, for each biomarker and subset, we identified the features that most effectively explained the variance (PCA-selected features, PCA-sel). Following this, we assessed whether the variance chosen through the initial feature selection exhibited a statistically significant linear relationship with the target biomarkers using Pearson's correlation and ANOVA tests as previously described. For feature selection and ML analyzes, the data were divided into training and testing subsets, with 25% of the data allocated to the test partition. Moreover, all subsets were standardized using the mean and standard deviation derived from the training data.

## 4 Machine Learning analyzes

ML analyzes were performed using the scikit-learn Machine Learning library in Python (Pedregosa et al., 2011). We trained several classical ML models using the four subsets of variables that, according to the PCA, best explained the variance of the dataset.

Dimensionality reduction helped to remove irrelevant, redundant, and noisy data, which in turn improved model performance and the intelligibility of the results. Model hyperparameters were fitted via five-fold cross-validation to ensure robustness. The models' effectiveness was assessed by calculating the Mean Absolute Error (MAE in pg/ml) across both the training and the test datasets. The MAE metric was preferred over the commonly used root mean square error as it provides a clearer and more direct measure of the average error, according to some studies (Willmott and Matsuura, 2005).

A Regression model based on the k-nearest neighbors (KNN) algorithm was trained, where the target prediction is based on local interpolation from the nearest neighbors in the training set. Additionally, we trained two Gaussian Process (GPs) models employing a radial basis function (RBF) kernel and a Matern 3/2 kernel. These GP models were optimized 20 times using the GPy framework in Python, with the optimal solution selected from these iterations. For ensemble learners utilizing bagging techniques, we optimized a Bagging Regressor (BR), a Random Forest Regressor (RFR), and an Extra Trees Regressor (ETR). With regards to boosting methods, we fitted three Gradient Boosting Regressors (GBRs) with distinct loss functions: least squares (LS), least absolute deviation (LAD), and a hybrid approach (Huber).

In terms of models with strong regularization policies, we fitted a linear regression with L2-norm regularization, known as Ridge Regression, and another with L1-norm regularization (Lasso). Furthermore, we trained four Support Vector Regressions (SVRs) using different kernels: linear (LIN), polynomial (POLY), radial basis function (RBF), and sigmoid (SIG).

## 5 Results

### 5.1 Demographic and clinical characteristics

A total of 61 subjects were recruited for this study. However, three subjects that did not possess PSG recordings and 9 whose biomarker measures were missing were excluded from the analysis, reducing the dataset to 49 subjects.

The population was equally distributed by gender (females were 50.8%), and the median age of the study population was 75.0 [72.0; 78.0] years. The Body Mass Index (BMI) median was 28.0 [24.4; 31.1]. The most frequently associated comorbidities were hypertension (63.9%), dyslipidemia (49.2%), heart disease (19.7%), and diabetes mellitus (16.4%). Concerning global cognitive dysfunction, the median MMSE score was 23.0 [21.0; 25.0]. Only a few patients reported symptoms indicative of poor sleep quality, such as insomnia, non-restorative sleep, nocturnal awakenings, and daytime drowsiness. The cohort was free from significant subjective daytime sleepiness, as indicated by a median score of 5.0 [3.0–8.0] on the Epworth Sleepiness Scale.

**Figure 2:**
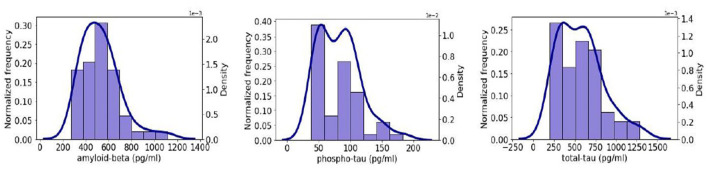
Histogram (purple) and density plots (dark blue) of the three biomarkers considered for the prediction on our dataset: **(left)** A*β*42; **(center)** p-tau; and **(right)** t-tau.

The PSG data indicated a high prevalence of OSA, with 26 (42,62%) patients. The median (IQR) AHI was 27.0 events/hour [15.4; 52.5]. The median ODI was 18.3 [12.6; 44.0] events/hour, and a median T90 was 11.9% [0.7; 7.2]. On average, participants slept (TST) 262.1 [203.5; 326.1] min. Sleep was highly fragmented, with a median arousals index (AI) value of 36.1 [23.2; 49.5] arousals per hour. In Figure 2, the density and histogram plots of the three studied biomarkers are shown. We observe that t-tau and p-tau shows a right skewed behavior while A*β*42 is closer to symmetric. Table 3 presents a summary of the clinical and demographic characteristics of the study population.

**Table 3:** Descriptive characteristics of the study population.

**Feature**	**Mean ±std**
**Sociodemographic data**	
Gender (women)	31 (50.8%)
Age (years)	75.0 [72.0; 78.0]
BMI, kg/m^2^	28.0 [24.4; 31.1]
**Comorbidities**	
Hypertension	39 (63.9%)
Diabetes mellitus	10 (16.4%)
Dyslipidemia	30 (49.2%)
Heart diseases	12 (19.7%)
Hypertension	3 (4.9%)
OSA	26 (42.6%)
**Alzheimer's disease parameters**	
Mini-mental state examination	23.0 [21.0; 25.0]
A*β*42 (pg/ml)	515.0 [404.25; 614.25]
t-tau (pg/ml)	555.0 [327.5; 712.0]
p-tau (pg/ml)	82.0 [52.0; 98.75]
**Polysomnographic parameters**	
Epworth sleepiness scale	5.0 [3.0; 8.0]
Time in bed (minutes)	420.1 [391.0; 446.1]
Total sleep time (minutes)	262.1 [203.5; 326.1]
Sleep efficiency (%)	66.4 [49.7; 79.4]
N1 stage (% TST)	11.96 [7.7; 18.4]
N2 stage (% TST)	23.1 [16.0; 35.1]
N3 stage (% TST)	13.6 [7.1; 22.4]
REM stage (% TST)	6.6 [3.3; 11.2]
REM sleep (% TST)	6.6 [3.3; 11.2]
Latency to N1 stage (minutes)	23.1 [11.9; 57.7]
Latency to REM sleep (minutes)	126.7 [85.1; 179.9]
AHI (events/hour)	27.0 [15.4; 52.5]
AI (events/hour)	36.1 [23.2; 49.5]
Mean SaO2 (%)	93.0 [92.0; 94.0]
CT90 (%)	1.9 [0.7; 7.2]
ODI (events/h)	18.3 [12.6; 44.0]

AHI, apnoea-hypopnea index; AI, arousal index; ApoE, apolipoprotein E; A*β*42, amyloid-beta protein; BMI, body mass index; ESS, Epworth sleepiness scale; IQR, interquartile range; ODI, oxygen-hemoglobin desaturation index; SaO2, oxygen-hemoglobin saturation; TST, total sleep time; CT90, percent of TST spent below 90% oxygen-hemoglobin desaturation; p-tau, phospho-tau; t-tau, total-tau.

### 5.2 Correlations between CSF biomarkers and selected variables of the four subsets

The statistical analysis conducted on various subsets of data revealed specific correlations between sleep-related variables and the levels of AD CSF biomarkers. The results of the statistical analyzes for the quantitative features identified as relevant by PCA are presented in Tables 4–6 for the CSF A*β*42, p-tau, and t-tau biomarkers, respectively. Similarly, the results for the categorical variables are shown in Tables 7–9.

**Table 4:** Pearson's correlation and the *p*-value between CSF A*β*42 biomarker and the quantitative variables that were considered relevant by the PCA algorithm.

**Variables**	**Correlation coefficient**	***p*-value**
**PSGVAR**		
EEGC3_A2_sk_N2	0.2630	0.0678
EEGO1_A2_sk_N1	0.3534	**0.0127** ^*^
EEGO1_A2_sp_N1	0.3285	**0.0211** ^*^
EEGO1_A2_sk_N2	0.3778	**0.0074** ^*^
EEGO1_A2_alpha_N2	0.4105	**0.0033** ^*^
EEGO1_A2_sp_N2	0.3563	**0.0119** ^*^
**SLEEPVAR**		
N^*o*^ Mixed Apnea	0.0826	0.5723
Mixed Apnea Index	0.1022	0.4843
N^*o*^ Arousals	0.1331	0.3619
Total Arousal Index	0.0802	0.5837
CT90	0.3129	**0.0322** ^*^
SpO_2_min	-0.1871	0.1978
**CLINVAR**		
Sleep (0–10)	-0.3853	**0.0062** ^*^
Sleep duration (Holidays)	-0.3981	**0.0046** ^*^

Values in bold denote statistically significant correlations: ^*^p < 0.05. alpha, alpha band; CT90, the percentage of TST during which oxygen saturation was < 90%; EEG, Electroencephalogram; EMG, Electromyogram; N1, N1 sleep stage; N2, N2 sleep stage; O2_A1, O2-A1 EEG channel; sk, skewness; sp, spindle; SpO_2_min, minimum oxygen saturation.

**Table 5:** Pearson's correlation and the *p*-value between CSF p-tau biomarker and the quantitative variables that were considered relevant by the PCA algorithm.

**Variables**	**Correlation coefficient**	***p*-value**
**PSGVAR**		
EEGF3_A2_theta_N2	0.2533	0.0790
EEGO2_A1_sk_N1	0.3077	**0.0314** ^*^
EEGO2_A1_sk_N2	0.3736	**0.0081** ^*^
EMGChin_ShanEnt_N3	0.2159	0.1541
EMG_Chin_max_N1	–0.3147	**0.0276** ^*^
EMGChin_max_N2	–0.2305	0.1109
EffortTHO_LempZiv_N2	0.3148	**0.0275** ^*^
Leg2_HDF_N2	0.1616	0.2671
Snore_HFD_N2	–0.1647	0.2579
**SLEEPVAR**		
Mixed Apnea Index	0.1256	0.3898
N^*o*^ Mixed Apnea	–0.1876	0.1966
Recording time	0.1439	0.3236
N3 Latency	0.2483	0.0852
SpO_2_min	0.1557	0.2850
**CLINVAR**		
Weight (Kg)	–0.2364	0.1057
Waist	–0.2506	0.0892
BMI	–0.2520	0.0839
Glucose	–0.1892	0.2242
Insulin	–0.2364	0.1316

Values in bold denote statistically significant correlations: ^*^p < 0.05. BMI, Body Mass Index; EEG, Electroencephalogram; EMG, Electromyogram; EffortTHO, Thoracic Effort; HFD, Higuchi Fractal Dimension; LempelZiv, Lempel Ziv; max, maximum value; N1, N1 sleep stage; N2, N2 sleep stage; N3, N3 sleep stage; O2_A1, O2-A1 EEG channel; ShanEnt, Shannon Entropy; sk, skewness; sp, spindle; SpO_2_min, minimum oxygen saturation.

**Table 6:** Pearson's correlation and the *p*-value between CSF t-tau biomarker and the quantitative variables that were considered relevant by the PCA algorithm.

**Variables**	**Correlation coefficient**	***p*-value**
**PSGVAR**		
EffortTHO_SampEnt_N2	0.3927	**0.0052** ^*^
EffortTHO_LempZiv_N2	0.4216	**0.0025** ^*^
**SLEEPVAR**		
Central apnea index	0.1724	0.2359
Recording time	0.1443	0.3225
Sleep efficiency	–0.1029	0.4815
N3 latency	0.2097	0.1479
N3 time	–0.1030	0.4811
% N3	–0.1407	0.3348
Time awake	0.1490	0.3067
**CLINVAR**		
Waist	–0.2420	0.1012
Insulin levels	–0.2409	0.1242

Values in bold denote statistically significant correlations: ^*^p < 0.05. EffortTHO, Thoracic Effort; LempelZiv, Lempel Ziv; max, maximum value; N3, N3 sleep stage; SampEnt, Sample Entropy.

**Table 7:** ANOVA *p*-value categorical features that were considered relevant by the PCA algorithm for predicting A*β*42 biomarker.

**CLINVAR variable**	***P*-value**
Physical activity	0.3624
Depression	0.2251
Epilepsy	0.2675
Pulmonary disease	0.1456
Non-restorative sleep	0.8501
Asphyxia crises	0.7328
Nocturia	0.6854
Headache	0.1863
Insomnia	0.1073
Cataplexy	0.1821
Real or vivid dreams	0.2154
Sleep interrupted by heartburn	**0.0296** ^*^
APOE4+	**0.0125** ^*^
Nap	0.6240

Values in bold indicate statistically significant correlations: ^*^p < 0.05.

**Table 8:** ANOVA *p*-value categorical features that were considered relevant by the PCA algorithm for predicting p-tau biomarkers.

**CLINVAR variable**	***P*-value**
Scholarship	**0.0136** ^*^
Depression	0.1557
Arterial hypertension	**0.0127** ^*^
Pulmonary disease	**0.0274** ^*^
Snore	0.1058
Asphyxia crises	0.8299
Insomnia	0.1476
Memory disorder	0.2441
Concentration disorder	0.1147
Cataplexy	0.1151
Grind teeth	0.2325
Nap	0.5223
APOE4+	0.1613

Values in bold indicate statistically significant correlations: ^*^p < 0.05.

**Table 9:** ANOVA *p*-value categorical features that were considered relevant by the PCA algorithm for predicting t-tau biomarkers.

**CLINVAR variable**	***p*-value**
Sex	0.3163
Arterial hypertension	0.0523
Heart disease	0.0905
Epilepsy	0.2752
Snore	**0.0149** ^*^
Memory disorder	0.5125
Cataplexy	0.1596
Grind teeth	0.2824
Nap	0.2119

Values in bold indicate statistically significant correlations: ^*^p < 0.05.

A*β*42: For the A*β*42 biomarker, poorer sleep quality (rho = -0.3853; *p* = 0.0062) and shorter sleep duration on holidays (rho = -0.3981; *p* = 0.0046) within the CLINVAR subset were linked to lower A*β*42 levels. Additionally, increased A*β*42 levels were found in individuals with sleep disturbances due to heartburn (*p* = 0.0296) and those carrying the ApoE genotype (*p* = 0.0123). In terms of polysomnography variables (PSGVAR), certain EEG characteristics during different sleep stages and while awake were positively correlated with A*β*42 levels such as the skewness in the EEG O1-A2 channel during the N1 (EEGO1_A2_sk_N1) and N2 (EEGO1_A2_sk_N2) sleep stages (rho = 0.3534, *p* = 0.0127 and rho = 0.3778, *p* = 0.0074, respectively). Notably, the only sleep variable (SLEEPVAR) that correlated positively with A*β*42 was the percentage of time spent with oxygen saturation below 90% during sleep (rho = 0.3129, *p* = 0.0322).

P-tau: Regarding the p-tau biomarker, increases were associated with specific EEG patterns during certain sleep stages and a negative correlation was found with the maximum chin electromyography (EMG) value during the N1 sleep stage (rho = –0.3147, *p* = 0.0276). In the clinical variables subset (CLINVAR), arterial hypertension (*p* = 0.0127), pulmonary diseases (*p* = 0.0274), and level of education (*p* = 0.0136) were significantly associated with p-tau levels.

T-tau: For the t-tau biomarker, significant correlations were identified with features derived from the thoracic effort channel such as the Lempel Ziv during N2 sleep stages (rho = 0.4216, *p* = 0.0025) and the Sample Entropy during N2 (rho = 0.3927, *p* = 0.0052), indicating a rise in t-tau levels. However, no significant relationships were observed with the general sleep variables, while the frequency of snoring was positively correlated with t-tau in the CLINVAR subset (*p* = 0.0149).

### 5.3 Regression models

**Figure 3:**
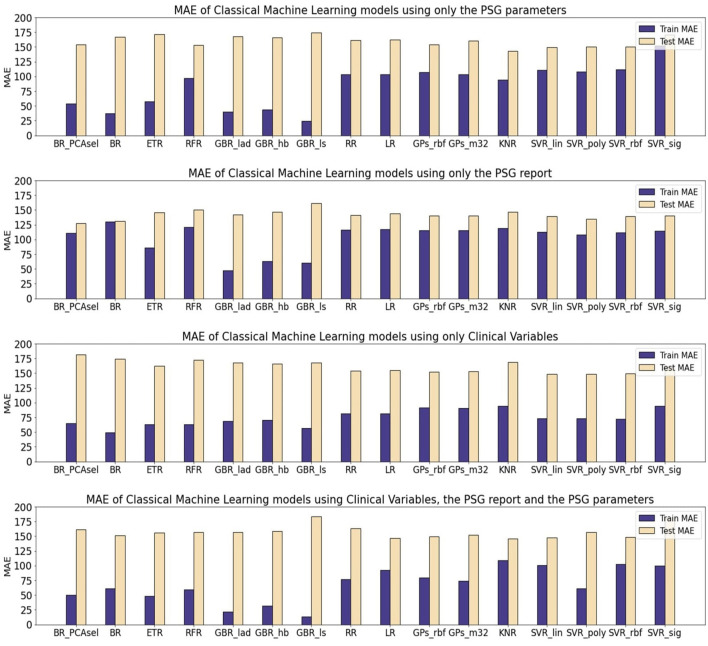
Mean average error (MAE) of Machine Learning (ML) models for the prediction of A*β*42. The results are shown for three different subsets. All of the models were optimized and evaluated using the PCA transformed subsets, except a Bagging Regressor that was trained using only the features considered by the PCA algorithm as relevant. The models evaluated were: a Bagging Regressor (BR), an Extra Trees Regressor (ETR), a Random Forest Regressor (RFR), a Gradient Boosting Regressor with a least absolute deviation loss (GBR_lad), a least squares loss (GBR_ls) and a Huber loss (GBR_hb), a Ridge Regression (RR), a Lasso Regression (LR), a Gaussian Process with a radial basis function kernel (GP_rbf) and a Matern 3/2 kernel (GP_m32), k-nearest neighbors regression (KNR), Support Vector Regression with a linear (SVR_lin), polynomial (SVR_poly), radial basis function (SVR_rbf), and sigmoid (SVR_sig) kernels.

**Figure 4:**
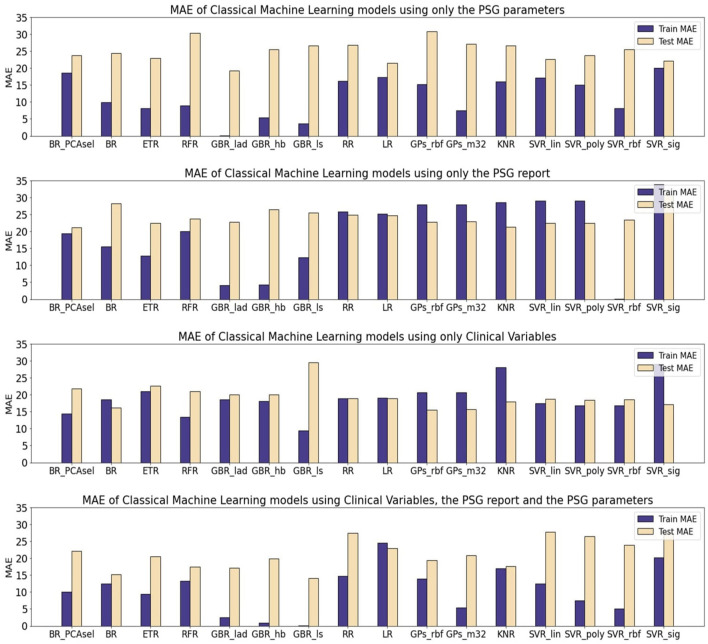
Mean average error (MAE) of Machine Learning (ML) models for the prediction of p-tau. The results are shown for three different subsets. All the models were optimized and evaluated using the PCA transformed subsets, except a Bagging Regressor that was trained using only the features considered by the PCA algorithm as relevant. The models evaluated were: a Bagging Regressor (BR), an Extra Trees Regressor (ETR), a Random Forest Regressor (RFR), a Gradient Boosting Regressor with a least absolute deviation loss (GBR_lad), a least squares loss (GBR_ls) and a Huber loss (GBR_hb), a Ridge Regression (RR), a Lasso Regression (LR), a Gaussian Process with a radial basis function kernel (GP_rbf) and a Matern 3/2 kernel (GP_m32), k-nearest neighbors regression (KNR), Support Vector Regression with a linear (SVR_lin), polynomial (SVR_poly), radial basis function (SVR_rbf), and sigmoid (SVR_sig) kernels.

**Figure 5:**
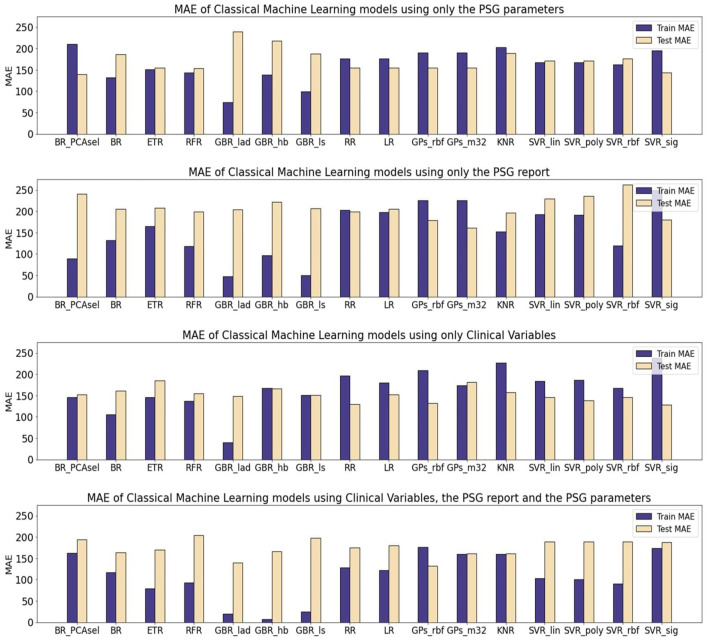
Mean average error (MAE) of Machine Learning (ML) models for the prediction of t-tau. The results are shown for three different subsets. All the models were optimized and evaluated using the PCA transformed subsets, except a Bagging Regressor that was trained using only the features considered by the PCA algorithm as relevant. The models evaluated were: a Bagging Regressor (BR), an Extra Trees Regressor (ETR), a Random Forest Regressor (RFR), a Gradient Boosting Regressor with a least absolute deviation loss (GBR_lad), a least squares loss (GBR_ls) and a Huber loss (GBR_hb), a Ridge Regression (RR), a Lasso Regression (LR), a Gaussian Process with a radial basis function kernel (GP_rbf) and a Matern 3/2 kernel (GP_m32), k-nearest neighbors regression (KNR), Support Vector Regression with a linear (SVR_lin), polynomial (SVR_poly), radial basis function (SVR_rbf), and sigmoid (SVR_sig) kernels.

Table 10 presents the mean absolute error (MAE) of each model family utilized in estimating the levels of CSF biomarkers. Figures 3–5 provide more comprehensive information.

**Table 10:** Mean and standard deviation of the mean average error (MAE) of Machine Learning (ML) models for the prediction of A*β*42, p-tau, and t-tau, biomarkers grouped by the family of models (EM, Ensemble models; RLR, Regularized Linear Regressions; GP, Gaussian Processes; KNR, regression based on k-nearest neighbors; SVR, Support Vector Regression).

	**A** *β* **42**							
	**CLINVAR**		**SLEEPVAR**		**PSGVAR**		**ALL**	
	**Train**	**Test**	**Train**	**Test**	**Train**	**Test**	**Train**	**Test**
EM	62.22 ± 7.33	170.43 ± 6.41	88.28 ± 32.71	143.5 ± 11.42	50.6 ± 23.21	164.88 ± 8.12	40.88 ± 18.72	160.67 ± 10.63
RLR	81.21 ± 0.16	154.33 ± 0.41	116.78 ± 1.12	142.35 ± 2.14	103.54 ± 0.59	161.63 ± 0.54	84.65 ± 10.74	154.97 ± 11.92
GP	91.08 ± 0.58	152.98 ± 0.41	115.44 ± 0.04	140.37 ± 0.05	105.29 ± 2.9	157 ± 4.37	77.02 ± 4.16	150.96 ± 1.91
KNR	93.86	168.58	119.59	147.13	94.67	142.83	109.03	146.15
SVR	78.52 ± 10.66	151.65 ± 5.68	111.73 ± 2.64	138.64 ± 2.4	120.73 ± 20.91	155.3 ± 10.32	91.12 ± 20.03	158.32 ± 14.96
	**p-tau**							
	**CLINVAR**		**SLEEPVAR**		**PSGVAR**		**ALL**	
	**Train**	**Test**	**Train**	**Test**	**Train**	**Test**	**Train**	**Test**
EM	16.22 ± 3.97	21.63 ± 4.06	12.65 ± 6.51	24.34 ± 2.47	7.81 ± 5.8	24.69 ± 3.42	6.95 ± 5.63	18.06 ± 2.9
RLR	19.01 ± 0.18	18.87 ± 0.02	25.49 ± 0.49	24.77 ± 0.05	16.75 ± 0.77	24.15 ± 3.74	19.64 ± 6.95	25.24 ± 3.15
GP	20.68 ± 0.02	15.61 ± 0.06	27.96 ± 0.02	22.93 ± 0.09	11.35 ± 5.4	29 ± 2.56	9.62 ± 6.03	20.16 ± 1.07
KNR	28.03	18.01	28.55	21.27	15.98	26.66	16.93	17.65
SVR	20.06 ± 5.97	18.21 ± 0.75	23.05 ± 15.47	24.8 ± 4.07	15.07 ± 5.09	23.5 ± 1.5	11.31 ± 6.72	26.39 ± 1.76
	**t-tau**							
	**CLINVAR**		**SLEEPVAR**		**PSGVAR**		**ALL**	
	**Train**	**Test**	**Train**	**Test**	**Train**	**Test**	**Train**	**Test**
EM	127.62 ± 42.83	159.9 ± 12.63	99.8 ± 42.75	212.37 ± 14.28	135.38 ± 43	182.84 ± 36.33	71.86 ± 57.53	176.69 ± 22.85
RLR	188.72 ± 12.03	140.95 ± 16.46	200.41 ± 3.55	202.34 ± 4.99	176.73 ± 0.05	154.43 ± 0.02	125.48 ± 4.63	177.74 ± 2.86
GP	191.49 ± 25.43	156.53 ± 34.57	225.73 ± 0.56	170.56 ± 12.81	190.18 ± 0.02	154.97 ± 0.19	168.32 ± 11.96	146.72 ± 21.1
KNR	226.55	158.10	152.89	196.31	203.38	189.49	159.87	161.65
SVR	193.99 ± 30.78	139.78 ± 8.45	188.53 ± 53.44	227.01 ± 34.48	172.85 ± 14.93	165.94 ± 15.26	116.96 ± 38.37	188.48 ± 0.6

We tried with four different datasets: CLINVAR, clinical variables; SLEEPVAR, conventional sleep parameters assessed by expert physicians; PSGVAR, Parameters that were computed over the PSG signals; ALL, the three previous ones combined.

For the A*β*42 biomarker, the test partition yielded higher MAE values across all models, regardless of the subject of data utilized. The performance in regression was comparable across different model families. Notably, using solely quantitative PSG signal parameters (PSGVAR), the training MAE was reduced for ensemble models (50.6 ± 23.21) compared to regularized regressions (103.54 ± 0.59), Gaussian Processes (GPs; 105.29 ± 2.91), or Support Vector Regression (SVR; 118.42 ± 20.91). The test MAE for ensemble models (164.88 ± 8.12), regularized regressions (161.63 ± 0.54), GPs (157 ± 4.37), and SVR (155.30 ± 10.32) showed minimal variation, with the ensemble models even registering higher values. When incorporating the ALL subset, which combines both PSGVAR and CLINVAR features, a similar test performance was observed with a lower training MAE compared to using only PSGVAR. However, employing solely clinical features (CLINVAR) resulted in diminished performance for the ensemble models, while the MAE for the other models slightly decreased, leading to generally comparable results across all models. Conversely, utilizing conventional sleep parameter reports (SLEEPVAR) achieved the lowest test MAE, with all models yielding values under 144. An individual assessment of each model performance (Figure 3) reveals that, for A*β*42 biomarker prediction, Gradient Boosting Regressors (GBRs) consistently outperformed other models. The most efficient model, the GBR employing the least absolute deviation loss (GBR_lad) trained with the SLEEPVAR subset, had the lowest training and test MAE (Train MEA: 47.54, Test MAE: 141.8).

For predicting p-tau levels (Figure 4), the lowest MAE values were achieved with the ALL subset and GBRs, particularly the GBR_ls, which demonstrated an MAE of 0.02 for training and 14.12 for testing. However, when applying this same subset (ALL) to optimize the rest of the ML models, there was a decline in performance for both training and testing partitions. This pattern was also observed in the PSGVAR subset, where the ensemble models outperformed other model families during training (7.81 ± 5.80), but their test performance did not significantly differ. Conversely, models using the CLINVAR and SLEEPVAR subsets generally underperformed compared to those using the ALL or PSGVAR subsets, as summarized in Table 10.

In the case of t-tau prediction, similar to p-tau, the lowest MAE was recorded with the ALL subset and GBRs. However, the GBRs using Huber loss function showed superior training performance (Train MAE: 7.33, Test MAE: 166.83), while the best test performance was achieved using the LAD loss functions (Train MAE: 19.55, Test MAE: 140.02), as shown in Figure 5. On the other hand, the highest test errors occurred with models trained solely on conventional sleep parameters (SLEEPVAR) evaluated by trained physicians. For instance, the ensemble models' test MAE using the SLEEPVAR subset was 212.37 ± 14.28, compared to 159.90 ± 12 (63) with the CLINVAR subset, as reported in Table 10. Conversely, for the training partition, a different pattern emerges, with the CLINVAR subset presenting a higher MAE (127.62 ± 42.83) compared to the SLEEPVAR subset ii (99.80 ± 42.75). Notably, the Gradient Boosting Regressors (GBRs) trained on the CLINVAR subset demonstrated lower MAE values, as depicted in Figure 5. Furthermore, models calibrated solely with the quantitative parameters derived from PSG recordings (PSGVAR) displayed performance closely paralleling those utilizing the CLINVAR subset, as indicated in Table 10.

The models that yielded the most accurate estimations for the levels of the three biomarkers were the GBRs, specifically employing a least absolute deviation (LAD) loss for A*β*42, a least squares (LS) loss for p-tau, and a Huber loss for t-tau. The alternative ML models, in addressing the scarcity of training data, employed regularization techniques to construct simpler models, which resulted in higher training errors. For all three biomarkers, minimal training errors were observed when utilizing the comprehensive ALL subset, though the test evaluations varied. In terms of test performance, for the A*β*42 biomarker, the lowest MAE was recorded with the SLEEPVAR subset, that is, the subset comprising only the standard PSG parameters.

## 6 Discussion

In this study, we trained various ML models to evaluate which combination of selected subsets of non-invasive variables could accurately predict CSF A*β*42, p-tau, and t-tau biomarker levels in a cohort of patients with mild-moderate AD(AD). The subsets included clinical variables (CLINVAR) previously established as relevant to AD pathogenesis, conventional PSG parameters (SLEEPVAR), quantitative PSG features (PSGVAR) derived from advanced signal analysis, and a combination of all these variables (ALL). Variable selection was performed using a range of statistical approaches, with an emphasis on those with the highest discriminating power. These newly curated subsets, containing only relevant variables, were then used as input for the models.

Moreover, we investigated the correlations between the selected relevant variables and the CSF biomarkers to determine the nature of their relationships, linear or otherwise. The Gradient Boosting Regressors (GBRs) emerged as the most effective models in estimating the levels of the three biomarkers. In summary, the lowest training errors for all three biomarkers were observed when employing the ALL subset, although the test evaluations showed differing results. Regarding test performance, for the A*β*42 biomarker, the lowest mean average error (MAE) was achieved using the subset consisting solely of conventional PSG parameters, which was thereby identified as the best predictor of the CSF A*β*42 levels. Furthermore, it was found that the combination of clinical variables, conventional PSG parameters, and quantitative PSG features most effectively predicted CSF p-tau and t-tau levels. Interestingly, not all the selected variables demonstrated linear relationships with the biomarkers in question.

### 6.1 Best data subset for CSF A*β*42 prediction

According to our ML models, the most significant variables for predicting CSF A*β*42 levels were standard PSG parameters (SLEEPVAR), which included CT90, minimum SaO2, the number of Mixed Apneas, Mixed Apnea Index, the number of arousals, and Total Arousal Index.

The influence of hypoxemia on CSF A*β*42 is not unexpected, as numerous studies have indicated that cerebral hypoxia can escalate amyloid deposition. This is achieved through both genetic and epigenetic modifications by influencing the expression levels of enzymes critical for protein synthesis and degradation (Lall et al., 2019). Furthermore, hypoxia has been linked to calcium dysregulation in neurons and glial cells. This occurs through the formation of calcium-permeable pores, disruption of glutamate transmission, malfunction of intracellular calcium stores, and neuroinflammation (Lall et al., 2019). More closely, while the relationship between SaO2 min and A*β*42 is not linear, our results revealed a positive correlation between CSF A*β*42 and CT90, surprisingly indicating that more severe hypoxemia is associated with higher CSF A*β*42 levels. This finding could be underpinned by the protective effect of hypoxic preconditioning (Shah et al., 2013), a phenomenon known to activate endogenous neuroprotective mechanisms in models of cerebral hypoxic and ischemic conditions (Wang et al., 2017). In line with our observations, animal studies have suggested that hypoxic preconditioning can mitigate memory impairment and A*β* pathology (Zhang et al., 2020). However, these findings need to be corroborated with alternative analytical approaches and through longitudinal follow-up of our cohort.

Additionally, our research indicates that the Mixed Apnea Index is another standard PSG parameter that is predictive of CSF A*β*42 levels, once again with low correlation coefficients. This highlights the importance of specifically characterizing respiratory events that compose the AHI to predict various outcomes and develop customized therapeutic strategies accurately. Incorporating both apneas and hypopneas into a single measure, such as the AHI, for diagnosing and evaluating the severity of OSA, implies that these events are equivalent in their clinical implications. However, the existing evidence is not univocal (Kulkas et al., 2017; Spector et al., 2019). Interestingly, recent research appears to support our hypothesis, showing that in a population with mild-to-moderate AD, hypopnea and apnea events are distinctively associated with sleep architecture, levels of pathological AD markers, and cognitive decline (Targa et al., 2023).

Similarly, the predictive role of the AI for CSF A*β*42 that we identified—despite the association not being linear—underscores the importance of detailed and personalized sleep analysis in early AD. Corroborating our findings, studies have demonstrated that arousals differ in their oscillatory composition and have various associations with early AD-related amyloid neuropathology and cognitive function (Chylinski et al., 2021). Future studies are essential to explore these observations further and to determine if there are causal relationships.

### 6.2 Best data subset for CSF p-tau

The most effective combination of feature groups for predicting CSF p-tau levels overall included quantitative PSG features, conventional PSG parameters, and clinical variables (ALL subset).

Regarding the role of quantitative PSG signal features (PSGVAR), our findings underscored the significance of the skewness of the EEG signal from the O1-A2 channel during the N1 and N2 sleep stages. We noted a significant positive linear correlation with p-tau levels.

Levels of p-tau have been shown in previous studies to correlate well with the spread of tau pathology from temporal regions to other areas of the brain. Tau pathology, more than amyloid, is closely associated with observed atrophy in MRI, hypometabolism in FDG-PET, and cognitive symptoms in AD (Jack et al., 2018). Additionally, tau pathology typically spreads from the temporal to more posterior structures, particularly the parietal regions (Masters et al., 2015). Consequently, we hypothesize that higher levels of p-tau may correlate with increased tau pathology in these posterior structures. Moreover, skewness, as a measure of asymmetry in the probability distribution of a signal (Xiang et al., 2020), may reflect this increased pathology, potentially resulting in more pronounced alterations in EEG signals from these regions. This could render the signal more unpredictable and with distinct spatial patterns, given the positive correlation between the two variables.

This discovery may represent one of the most significant and unforeseen findings of our study, suggesting specific quantitative PSG features, such as sleep EEG, could serve as potential early biomarkers of AD. Prior research has supported the potential of EEG recordings for the early detection of AD. Furthermore, in line with our findings, other studies (Jeong, 2004; Ghorbanian et al., 2015; Liu et al., 2016) have indicated that a characteristic EEG abnormality in AD patients is a generalized slowing of the rhythms and a reduction in complexity across various brain regions. Interestingly, these EEG abnormalities in AD have been correlated with disease severity, as they directly reflect the anatomical and functional changes in the cerebral cortex that are affected by the disease (Kowalski et al., 2001).

Among the conventional PSG variables (SLEEPVAR), we find it noteworthy to highlight that our models selected minimum SaO2 as one of the parameters predictive of CSF p-tau levels, suggesting that more severe hypoxemia may lead to an increase in CSF p-tau levels, given the positive relationship. This finding aligns with previous results indicating that hypoxia triggers tau hyperphosphorylation and memory deficits in rats (Zhang et al., 2014).

Besides, our results regarding the clinical variables subset (CLINVAR) appear to corroborate existing evidence that arterial hypertension is a statistical predictor of p-tau accumulation, in line with previous findings, suggesting that hypertension may elevate the risk for AD (Lennon et al., 2021). Meanwhile, our data also indicate that metabolic clinical markers, including BMI, waist circumference, and serum insulin and glucose levels, are implicated in the prediction of p-tau protein levels in the CSF in AD, as explored in previous studies (Gonçalves et al., 2019; Lee et al., 2023). However, in our study, these relationships did not follow a linear pattern.

### 6.3 Best data subset for CSF t-tau

As observed for the prediction of CSF p-tau, the ALL subset also emerged as the best subset of variables for CSF t-tau levels.

Interestingly, regarding the PSGVAR subset, we found that the Lempel-Ziv and Sample Entropy—both of which are measures of signal complexity (Grassberger and Procaccia, 1983)—are pertinent to predict t-tau levels. Our results indicated that the complexity of the thoracic effort signals, increases with the levels of t-tau during NREM sleep.

Increased complexity of the thoracic effort signal may be associated with OSA syndrome, which is highly prevalent in our study population (Gaeta et al., 2020). It is known that increased respiratory effort against collapsed airways leads to elevated intrathoracic disturbances (Farré et al., 2004; Sánchez-de-la Torre et al., 2013). Consequently, a higher complexity in the thoracic effort signal may imply a more severe manifestation of OSA, thus, the positive correlation between Lempel-Ziv complexity and Sample Entropy of thoracic effort and tau CSF biomarkers could indicate an indirect link between OSA syndrome and the advancement of AD tau pathology. However, we did not find any correlation between t-tau and the AIH, affirming the hypothesis that a more precise definition of sleep-related breathing disorders in AD is necessary, as previously discussed (Targa et al., 2023).

Several mechanisms have been proposed to explain this phenomenon (Andrade et al., 2018). Previous investigations suggest that the effort to breathe against a collapsed upper airway during obstructive sleep apnea episodes may cause repetitive high-pressure fluctuations, potentially impacting the glymphatic system, thereby altering the concentration of CSF metabolites and biomarkers (Andrade et al., 2018). The glymphatic system operates optimally during SWA, facilitating the removal of A*β* and other metabolites from the interstitial space. Studies conducted with mouse models of AD have demonstrated that sleep deprivation acutely increases soluble A*β* levels and promotes chronic amyloid deposition (Ju et al., 2017). In human subjects, sleep deprivation has been observed to elevate cerebrospinal fluid (CSF) A*β* levels. Consequently, it has been theorized that frequent arousals in obstructive sleep apnea (OSA) contribute to reduced SWA, leading to elevated A*β* levels and ultimately increasing A*β* aggregation into amyloid, thereby heightening the risk of AD (Ju et al., 2016). Furthermore, persistent sleep fragmentation resulting from OSA disrupts CSF-ISF (Interstitial Spinal Fluid) exchange, leading to an accumulation of A*β* (Ju et al., 2016).

Nonetheless, contradictory findings have been documented in the literature. For example, Ju et al. (2016) observed lower concentrations of A*β*42, tau, and other derived metabolites in the CSF of patients with OSA. However, they noted that total protein levels, which are primarily derived from blood albumin, did not decrease in patients with severe OSA compared to control subjects (Ju et al., 2016), suggesting that glymphatic clearance processes may be compromised in OSA (Aspelund et al., 2015). This discrepancy underscores the need for future research that includes larger populations to compare OSA patients with non-OSA individuals.

We hypothesize that akin to the effect on CSF amyloid, the increased intrathoracic and intracranial pressures induced by OSA acutely and repeatedly may hinder the circulation of brain metabolites from ISF into CSF, thereby fostering tau accumulation. Our hypothesis is supported by prior studies revealing compromised CSF-ISF exchange polarization in a mouse model of tauopathy, indicating the potential of this clearance pathway to worsen or even initiate pathogenic tau accumulation (Harrison et al., 2020). Comprehensive analyzes are required.

Moreover, regarding the predictive value of the conventional PSG parameter (SLEEPVAR subset), our findings concur with earlier research, which has shown a negative association between tau pathology and time spent in N3 sleep stage. Remarkably, SWA during N3 sleep is widely reported to be disrupted in AD (Mander et al., 2015). Furthermore, consistent with our findings, has been demonstrated an inverse correlation between SWA and the AD tau pathology, as evidenced by PET imaging and CSF tau biomarker levels in the early stages of the disease (Lucey et al., 2019).

On the other hand, the results concerning the CLINVAR subset, while not displaying a significant linear correlation, overlap with those observed for p-tau, confirming the importance of metabolic measures, particularly insulin levels, in tau pathology. Insulin, a key regulator of glucose homeostasis and metabolism, plays a critical role in various brain functions, including synaptic plasticity, learning, and memory. Besides its traditional function as a microtubule-stabilizing protein, tau also serves as a scaffold protein interacting with components of the brain's insulin signaling pathway (Gonçalves et al., 2019). Impairment in brain insulin signaling has been consistently linked to cognitive decline in animal models and humans. Insoluble fractions of post-mortem AD and other tauopathies have revealed hyperphosphorylated tau-containing neurons with accumulated insulin oligomers (Gonçalves et al., 2019). While the exact influence of tau pathology on insulin signaling remains unclear, it's established that insulin resistance can lead to tau hyperphosphorylation and cognitive decline in both human and animal models (Gonçalves et al., 2019). However, the negative relationship between insulin serum levels and CSF t-tau we observed, remains to be more extensively investigated.

### 6.4 Related works

In light of the existing body of research, our study is among the first to evaluate a variety of ML models trained to non-invasively predict AD neuropathology by integrating sleep data, clinical variables crucial for AD development, conventional PSG parameters, and a broad spectrum of quantitative signal features from sleep data. Our approach stands out by employing a multi-domain quantitative signal feature analysis, encompassing both linear and non-linear aspects, which extends beyond the conventional spectral features typically utilized in prior studies. Additionally, our analysis encompasses the entirety of PSG signals and is not limited solely to EEG data. The landscape of ML applications in AD diagnosis and progression encompasses a diverse range of techniques and input variables. To illustrate, Table 11 presents a summary of pertinent studies, highlighting the ML techniques and proposed biomarkers at various stages of AD diagnosis. Key variables such as educational level, professional experience, social engagement, dietary habits, APOE genotype, and age have been identified as significant predictors for the conversion to MCI in a substantial elderly cohort over a 5-year timeframe, utilizing Random Forest and permutation-based Machine Learning methods (Gómez-Ramírez et al., 2020).

**Table 11:** Relevant studies on biomarkers and Machine Learning (ML) models of Alzheimer's disease (AD).

**References**	**Proposed AD biomarkers/ input variables**	**Machine Learning models**	**Results**	**Normal cognition/ Alzheimer's disease stage**
Gómez-Raḿırez et al. (2020)	Clinical variables	Random Forest (RF) Permutation-based Machine Learning (PBML)	Educational level, working experience, social life, subjective cognitive decline, diet rich in sweets, APOE and age have been selected as some of the most important variables for MCI conversion in a large elderly population, using RF that showed a high predictive performance with an accuracy of 0.851	Conversion from NC to MCI
Bonfa et al. (2023)	Sleep disturbances	Random Tree classifiers Logistic regression K-nearest neighbors (KNN)	Based on Random Tree classifiers, the best model predicted a 70% accuracy change from MCI to AD, with caregiver distress, sleep disturbance frequency, and excessive daytime sleepiness playing significant roles. Logistic regression and KNN models predicted with 60% accuracy re-conversion from MCI to NC, with caregiver distress, sleep disturbance frequency, and OSA most pronounced among males.	Conversion from NC to MCI and from MCI to other stages of AD
Nyholm et al. (2024)	Sleep and clinical variables	Gradient Boosting (GB) Logistic regression Gaussian naive Bayes (GNB) Random Forest (RF) Support Vector Machine (SVM)	GB resulted the most accurate method, with 92.9% accuracy, 0.926 f1-score, 0.974 ROC AUC, and a Brier score of 0.056. Sleeping more than 2 h per day, gender, education level, age, waking up throughout the night, and snoring were the variables with the greatest feature importance across all ML algorithms.	Age > 60 years from the Swedish National Study on Aging and Care (SNAC) including NC, MCI and moderate dementia
Abate et al. (2020)	Clinical variables and plasma p53	Regression Tree (RT)	Plasma p53 was found more relevant for identifying accurately classify (AUC = 0.92) A*β*^+^/amnestic Mild Cognitive Impairment (aMCI) patients who will develop AD.	MCI
Chang et al. (2021)	Plasma D-glutamate	Support Vector Machine (SVM) Logistic regression Random Forest (RF) Naïve Bayes	The naïve Bayes model and RF model were the best models for determining MCI and AD susceptibility based on D-glutamate, respectively (area under the receiver operating characteristic curve: 0.8207 and 0.7900; sensitivity: 0.8438 and 0.6997; and specificity = 0.8158 and 0.9188, respectively).	MCI Others stages of AD
Jo et al. (2020)	Tau- Positron Emission Tomography (PET)	Convolutional Neural Network (CNN) Layer-wise relevance propagation (LRP) algorithm	Deep learning-based classification model framework combining 3D CNN and LRP algorithms of AD from NC yielded an average accuracy of 90.8%	Alzheimer's Disease Neuroimaging Initiative (ADNI) cohort including HC, MCI and others AD stages
Popuri et al. (2020)	Magnetic Resonance Images (MRI) t-tau A*β*1-42 CSF	Ensemble-learning (Multi-Kernel classifier, Variational Bayes Probabilistic Multi-Kernel Learning)	MDART (MRI-based on Dementia Alzheimer's type DAT) score achieved a classification performance on stable vs. progressive MCI groups with an AUC of 0.81 for TTC of 6 months and 0.73 for TTC of up to 7 years	NC MCI and others AD stages
Pirrone et al. (2022)	Resting-state EEG power spectral density features	Decision Trees (DT) Support Vector Machines (SVM) K-nearest neighbor (KNN)	K-NN was the best classification algorithm as concerning the accuracy reaching 97, 95, and 83% accuracy when considering binary classifications (HC vs. AD, HC vs. MCI, and MCI vs. AD) and an accuracy of 75% when dealing with the three classes (HC vs. AD vs. MCI).	HC, MCI, others stage AD
Kim et al. (2023)	Resting-state EEG power spectral density features	Support Vector Machine (SVM) Logistic regression K-nearest neighbors (KNN) Gaussian Naive Bayes (GNB) Random Forest (RF) AdaBoost (Ada) GBMBoost (GBM) XGBoost (XGB)	The best model demonstrated 90.9% sensitivity, 76.7% specificity, and 82.9% accuracy in MCI+SCD (33 A*β*^+^, 43 A*β*^−^). Limited to SCD, 92.3% sensitivity, 75.0% specificity, and 81.1% accuracy (13 A*β*^+^, 24 A*β*^−^). 90% sensitivity, 78.9% specificity, and 84.6% accuracy for MCI (20 A*β*^+^, 19 A*β*^−^).	Subjective cognitive decline (SCD), MCI
Kim et al. (2021)	Resting-state EEG power spectral density features	Multi-Model Ensembles Genetic Algorithms Support Vector Machine (SVM)	Genetic Algorithm Heuristic achieved 85.7% sensitivity, 89.3% specificity, and 88.6% accuracy in (SCD) amyloid positive/negative classification, and 83.3% sensitivity, 85.7% specificity, and 84.6% accuracy in MCI amyloid positive/negative classification.	Subjective cognitive decline (SCD), MCI
Gaubert et al. (2021)	Clinical variables Resting-state EEG power spectral density features (qEEG) Magnetic Resonance Imaging (MRI)	Random Forest (RF) Logistic regression Support Vector Machine (SVM)	qEEG was the strongest predictor of neurodegeneration, with 82% negative predictive value (NPV), 38% positive predictive value (PPV), 77% specificity and 45% sensitivity. The combination of demographic, neuropsychological data, ApoEϵ4, and MRI hippocampal volumetry most strongly predicted amyloid (80% NPV, 41% PPV, 70% specificity, 58% sensitivity) and most strongly predicted decline to prodromal AD at 5 years (97% NPV, 14% PPV, 83% specificity, and 50% sensitivity).	French INSIGHT-preAD cohort, which includes baseline data cognitively normal individuals, between 70 and 85 years old, with subjective memory complaints but unimpaired cognition, prodromal AD
Geng et al. (2022)	Sleep slow-waves and spindles data, spectral and complexity features from sleep EEG.	Support Vector Machine (SVM) Gate Recurrent Unit network techniques (GRU)	The MCI classification accuracy of the GRU network based on features extracted from sleep EEG was the highest at 93.46%.	MCI, HC
Khosroazad et al. (2023)	Power Spectral Density features from PSG	Neural Networks (NN) Kernel algorithms	Time-Lag (TL), extracted from high-frequency movements and respiratory alterations during sleep early detect MCI in AD, resulting in high sensitivity (86.75% for NN and 65% for Kernel), specificity (89.25 and 100%), and accuracy (88 and 82.5%).	NC, MCI

Ada, AdaBoost; AD, Alzheimer's Disease; AUC, Area under curve; CNN, Convolutional Neural Network; DT, Decision Trees; EEG, Electroencephalography; GNB, Gaussian Naive Bayes; GRU, Gate Recurrent Unit network techniques; GBM, GBMBoost; GB, Gradient Boosting; KNN, K-nearest neighbors; LRP algorithm, Layer-wise relevance propagation; MRI, Magnetic Resonance Images; MCI, Mild Cognitive Impairment; NPV, Negative predictive value; NN, Neural Networks; NC, normal cognition; PBML, Permutation-based Machine Learning; PET, Positron Emission Tomography; PSG, Polysomnography; PPV, Positive predictive value; RF, Random Forest; RT, Regression Tree; SCD, Subjective cognitive decline; SVM, Support Vector Machine; SNAC, Swedish National Study on Aging and Care; TL, Time-Lag; XGB, XGBoost.

Recent studies have increasingly focused on sleep-related variables, parameters, and quantitative signal features, utilizing techniques. However, many of these studies did not simultaneously incorporated various types of sleep measures, where not conducted explicitly on AD populations, or limited their quantitative signal analysis to resting state EEG data. For example, one study employed advanced ML models to assess the impact of sleep disturbances on the development of AD and the transition from normal cognitive (NC) functioning to MCI. Employing Random Tree classifiers, the most effective model achieved a 70% accuracy rate in predicting the transition from MCI to AD, with factors such as caregiver distress, frequency of sleep disturbances, and excessive daytime sleepiness playing significant roles. Logistic regression and KNN models predicted with 60% accuracy re-conversion from MCI to NC, with caregiver distress, sleep disturbance frequency, and OSA most pronounced among males (Bonfa et al., 2023).

Moreover, research has confirmed the link between dementia and sleep disturbance, with gradient boosting emerging as the most accurate method, boasting a 92.9% accuracy rate, and F1-score of 0.926, and ROC AUC of 0.974, and a Brier score of 0.056. The relevant factors differed amongst ML algorithms, while sleeping more than 2 h per day, gender, education level, age, waking up throughout the night, and snoring were the variables with the greatest feature importance across all ML algorithms (Nyholm et al., 2024).

Interestingly, Kim et al. (2021) developed a spectral qEEG-ML algorithm to predict AD defined by A*β*-PET positivity among patients with subjective cognitive decline (SCD) and MCI. Genetic Algorithm Heuristic achieved 85.7% sensitivity, 89.3% specificity, and 88.6% accuracy in SCD amyloid positive/negative classification, and 83.3% sensitivity, 85.7% specificity, and 84.6% accuracy in MCI amyloid positive/negative classification, confirming previous results. The authors used other types of EEG-ML algorithms to detect brain A*β* pathology validated with A*β* PET. The best model demonstrated 90.9% sensitivity, 76.7% specificity, and 82.9% accuracy in MCI + SCD (33 A*β*^+^, 43 A*β*^−^), suggesting that qEEG could serve as a promising biomarker for A*β* amyloid pathology (Kim et al., 2023).

Furthermore, Gaubert et al. (2021) evaluated the efficacy of an ML approach in achieving early AD diagnosis based on A*β*-PET and MRI measurement, introducing a multimodal non-invasive biomarker strategy. Importantly, qEEG was the strongest predictor of neurodegeneration, with 82% negative predictive value, 38% positive predictive value, 77% specificity, and 45% sensitivity. Besides the combination of demographic, neuropsychological data, ApoEϵ4 and MRI hippocampal volumetry most strongly predicted amyloid (80% NPV, 41% PPV, 70% specificity, and 58% sensitivity).

Remarkably, innovative research utilizing Support Vector Machine classifiers and Gate Recurrent Unit network techniques has demonstrated the potential of sleep EEG data, including slow waves and spindles, to distinguish MCI from HC with high accuracy (Geng et al., 2022). The MCI classification accuracy of the GRU network based on features extracted from sleep EEG was the highest at 93.46%. Their experimental findings demonstrate that, as compared to awake EEG, sleep EEG can give more helpful information for identifying MCI and HC.

Additionally, pioneering work has proposed a novel diagnostic approach for MCI in AD, combining sleep-related movements with advanced signal processing and ML techniques (Khosroazad et al., 2023). This approach, which explores the relationship between high-frequency movements and respiratory changes during sleep, highlights the potential of advanced quantitative sleep signal feature analyzes in a multimodal ML setting for early detection of AD neuropathology. The approach employs Neural Networks and Kernel algorithms, resulting in high sensitivity (86.75% for NN and 65% for Kernel), specificity (89.25 and 100%), and accuracy (88 and 82.5%) for early detection of MCI in AD.

Despite these advancements, the extensive exploration of advanced quantitative sleep signal features to no invasively detect the AD neuropathology within a multimodal ML framework remains a promising area for future research.

### 6.5 Strengths and limitations of the study

To our knowledge, our study for the first time integrated clinical variables, conventional PSG parameters, and quantitative PSG signal features for the non-invasive prediction of core CSF biomarkers of AD using various ML models.

This study is novel in that it applies computational engineering to extract a wide range of quantitative signal features from thoroughly preprocessed PSG signals, identifying potential biomarkers for the early detection of AD. To date, no research has delineated the relationship between these specific quantitative PSG features and AD-related CSF biomarkers of neurodegeneration, suggesting that sleep qEEG could demonstrate a topographic specificity in their associations. Our approach involved working with spectral, time-domain, and non-linear parameters directly extracted from electrophysiological signals, indicating that PSG recordings may contain additional crucial information for the diagnosis of AD.

A significant aspect of this research is the rigorous cross-validation ML methodology employed. We trained multiple ML models to determine which could deliver the best performance for our outcomes. The models most effective at addressing the overfitting issue were Gradient Boosting Regressors, utilizing a least absolute deviation loss function for A*β*42, a least squares loss for p-tau and a Huber loss for t-tau. The other optimized ML models generally handled the scarcity of training data by using regularization to construct simpler models that accept higher training errors.

Moreover, in assessing correlations between the selected variables and the CSF biomarkers, we found that not all variables showed significant linear associations with the targets. This demonstrates how ML facilitates the exploration of non-linear relationships that may not be detectable using conventional statistical methods, in addition to providing several other advantages like as fault tolerance and real-time operation, making them suitable for complex applications (Chang et al., 2021). In the realm of modeling, linearity denotes a system or model's characteristic where the output varies directly with the input, while non-linearity suggests a more intricate relationship between input and output, eluding simple linear expressions attainable through conventional statistical means. ML prioritizes precise predictions, while traditional statistical models concentrate on uncovering interrelations among variables. ML offers advantages in flexibility and scalability over conventional statistical techniques, rendering it suitable for diverse tasks such as diagnosis, classification, and survival prognosis. A fundamental disparity between ML and traditional statistical methods lies in their primary objectives: the former emphasizes predictive accuracy, while the latter focuses on deducing relationships among variables. Additionally, ML adeptly addresses interactions, which are difficult to investigate with traditional statistical methods that primarily handle interactions between the principal determinant and single potential confounders (Rajula et al., 2020).

We acknowledge the limitations of our study, which are important to consider when interpreting the results. One of the primary limitations is the reliance on a relatively small sample size of 49 participants, which may limit the generalizability of our findings. Furthermore, ethical considerations prevented us from conducting a comparative analysis with a control group. The absence of neuroimaging assessments, such as MRI, which are known to correlate with disease progression and serve as critical predictive markers for AD in patients with MCI (Jack et al., 2018), is another limitation that merits attention. Moreover, we recognize a notable limitation in our study, as patients were included solely based on the 2011 NIA-AA clinical criteria (McKhann et al., 2011), without considering the updated 2018 biological criteria (Jack et al., 2018), due to the trial registration in 2016. This approach may not fully capture the evolving understanding of the disease, where biomarkers exhibit changes years before symptom onset. The presence of normal biomarker levels, especially amyloid pathology, in individuals with dementia suggests the potential for an alternative diagnosis (Jack et al., 2018). Given these limitations, we recommend a cautious interpretation of our results, although we believe they contribute valuable insights.

The models that excelled in predicting the levels of the three biomarkers were the Gradient Boosting Regressors, which utilized a LAD loss for A*β*42, an LS loss for p-tau, and a Huber loss for t-tau. These are among the most common loss functions for regression challenges. The distinctions between them are as follows:

1. LAD loss is less sensitive to outliers but backs a closed-form solution due to its non-continuous derivatives;
2. LS loss, on the other hand, penalizes large deviations more severely, making it less robust to outliers, yet it tends to yield more stable solutions (Natekin and Knoll, 2013);
3. The Huber loss integrates aspects of both LAD and LS losses (Natekin and Knoll, 2013).

For instance, in predicting A*β*42 levels, outliers could be disregarded with relative safety. In contrast, outliers played a more critical role in optimizing the models for the tau-related biomarkers. Examining Figure 1 reveals that p-tau and t-tau have similar density distributions. Additionally, boosting techniques are generally recognized for their low bias, which is the discrepancy between the average prediction of the model and the actual value. Put simply, a high-bias model tends to be overly simplistic and does not fit the training data well, leading to significant errors in both the training and the test datasets. However, it is essential to consider that a low-bias model often has high variance, increasing the risk of overfitting the training data.

Thus, despite the Gradient Boosting Regressors achieving some of the lowest training errors, their MAEs on the test set were still considerable. This suggests that, even with cross-validation to fine-tune the models for an optimal balance in the bias-variance trade-off, the models may still not generalize well beyond the training data. In terms of ensemble models employing bagging techniques, they typically displayed test errors comparable to those of boosting methods but with increased training errors. Models such as the Bagging Regressor (BR), Random Forest Regressor (RFR), and Extra Trees Regressor (ETR) function by aggregating the decisions for T individual decision trees, each trained on a bootstrapped subset of the data. The final prediction is the majority vote across these T trees. This approach reduces the variance associated with single decision trees, resulting in a model that is less prone to overfitting and demonstrates improved generalization. Furthermore, decision trees, like those in a random forest, provide insight into the importance of features during training. However, the application of PCA for dimensionality reduction, while preventing noise overfitting, obscures the interpretability of model decisions. Other models generally address overfitting by deliberately allowing higher training errors, thereby enhancing their generalization capabilities to new data at the expense of complexity.

Gaussian Process (GP) models, in contrast, exhibited higher training errors than test errors, signifying potential underfitting. This could indicate that the selected kernel was insufficient in capturing the complexity of the data, potentially necessitating a more sophisticated kernel for optimization. Although GPs can assess feature importance through kernel length scales, this interpretability is lost when PCA is applied. Support Vector Regressors (SVRs) struck a better balance between training and test errors, often with training errors marginally exceeding those of other models. The K-Nearest Neighbors Regressors (KNR) also managed the bias-variance trade-off effectively, sometimes with a training error even surpassing the test error.

Regarding the analysis of the three biomarkers, the lowest training errors were associated with the “ALL” subset (incorporating clinical, PSG, and quantitative PSG-derived variables), though test evaluations varied. This could be attributed to the retention of a greater number of principal components for the “ALL” subset compared to others, potentially facilitating overfitting. Additionally, the principal components of the “ALL” subset represent linear combinations of a broader range of features, thus encapsulating more detailed information about each sample. For the A*β*42 biomarker, the “SLEEPVAR” subset—comprising solely sleep parameters evaluated by experts-yielded the lowest MAE during testing. However, it is important to note that none of the models achieved optimal performance. Therefore, while the findings are significant, they should be interpreted with caution. To ensure the robustness of our findings across various populations, we advocate for subsequent validation studies to be conducted with independent cohorts of cognitively normal subjects. Additionally, enhancing the predictive accuracy of our models through incorporating data from multiple preclinical cohorts and applying longitudinal, multimodal measures within a nested cross-validation framework represents a promising avenue for future research.

An intriguing prospect for further investigation is the integration of neuropsychological assessments and MRI data as well as blood biomarkers into our analytical framework. This approach would allow for a thorough longitudinal evaluation of the clinical progression toward prodromal AD, leading to a deeper comprehension of neurodegenerative mechanisms. Additionally, it would improve the predictive accuracy of our models and provide less invasive methods for early AD detection. Upon validation of our initial results, a critical next step would be to identify the most predictive set of features for neurodegeneration, using performance metrics as a guide. Advanced artificial intelligence techniques could then be employed to determine the ability of these features to differentiate between AD, MCI and HC groups. Moreover, the exploration of automated analysis of PSG recordings through neural network integration into our model offers a promising direction. Such automation could potentially eliminate the need for manual annotation by enabling the automatic detection of artifacts and sleep stages.

## 7 Conclusions

This study highlights the potential of ML to assess asymptomatic individuals at risk for AD through the analysis of non-invasive and cost-effective biomarkers, underscoring the ability of ML to uncover complex non-linear relationships within intricate datasets, that may elude traditional statistical methods. This could offer supplementary insights alongside other biomarkers, hinting at AD pathology in asymptomatic individuals, or functioning as an additional diagnostic tool for those ineligibles for CSF biomarkers determination.

Ours results also suggest that relying solely on a single type of biomarker may not suffice for a reliable AD early detection. Notably, we emphasize the importance of specific quantitative PSG signal features such as EEG skewness, Lempel Ziv and Sample Entropy of thoracic effort signals, as reliable markers for predicting neurodegeneration, along with conventional PSG parameters. The utilization of portable PSG devices may establish a groundwork for their utilization in clinical environments.

The demonstration of the viability of these innovative approaches underlines their potential contribution to the early diagnosis of AD, particularly through the prediction of core CSF biomarkers and the exploration of their relationships with sleep patterns.

## Data availability statement

The original contributions presented in the study are included in the article/Supplementary material, further inquiries can be directed to the corresponding author.

## Ethics statement

The studies involving humans were approved by the study adhered to the principles of the Declaration of Helsinki and received approval from the Ethics Committee of Hospital Arnau de Vilanova de Lleida (CE-1218). The studies were conducted in accordance with the local legislation and institutional requirements. The participants provided their written informed consent to participate in this study.

## Author contributions

AG: Conceptualization, Data curation, Investigation, Methodology, Visualization, Writing – original draft, Writing – review & editing. MQ-L: Conceptualization, Formal analysis, Validation, Visualization, Writing – original draft, Writing – review & editing. FB: Conceptualization, Funding acquisition, Investigation, Methodology, Project administration, Resources, Supervision, Writing – review & editing. RV: Data curation, Writing – review & editing. MP: Data curation, Writing – review & editing. OM: Data curation, Writing – review & editing. MS: Conceptualization, Investigation, Methodology, Writing – review & editing. AM-B: Conceptualization, Funding acquisition, Resources, Supervision, Validation, Writing – original draft, Writing – review & editing. GP-R: Conceptualization, Funding acquisition, Project administration, Resources, Supervision, Validation, Writing – review & editing.
