# Supplementary material for: Predicting Alzheimer's disease CSF core biomarkers: a multimodal Machine Learning approach
Source: Front Aging Neurosci. 2024 Jun 26;16:1369545. doi: 10.3389/fnagi.2024.1369545 (PMC11233742; doi:10.3389/fnagi.2024.1369545)
Supplement: Supplementary file 1 [file Data_Sheet_1.pdf]

Supplementary Material

Table S1. Overview of overall sociodemographic, anthropometric, and clinical variables.

**Type of variables**	**Variables**
Sociodemographic	Sex<br>Age<br>Smoker (0-No; 1-Yes; 2- Former smoker, (6 months without smoking)<br>Alcohol (0-No; 1-Yes; 2- Former alcoholic)<br>Physical activity (0-Sedentary; 1-Moderate; 2-Active)<br>Scholarship (0-No studies; 1-Primary; 2-Secondary; 3-University)
Diseases	Arterial Hypertension (HTA) (0-No; 1-Yes; 2-Resistant)<br>Heart disease (0-No; 1-Yes)<br>Ictus (0-No; 1-Yes)<br>Epilepsy (0-No; 1-Yes)<br>Pulmonary Disease (0-No; 1-COPD; 2-Asthma; 3-PE; 4-Pneumonia; 5-Others)<br>Diabetes mellitus (DM) (0-No; 1-Yes)<br>Neoplasm (0-No; 1-Yes)<br>Memory disorder (0-No; 1-Sometimes; 2-Frequently; 3-Always)<br>Concentration disorder (0-No; 1-Sometimes; 2-Frequently; 3-Always)<br>Depression (0-No; 1-Yes)<br>Anxiety (0-No; 1-Yes)
Sleep	Sleep (0-10)<br>EPWORTH Sleepiness Scale (1-23)<br>Snore (0-No; 1-Sometimes; 2-Frequently; 3-Always)<br>Nonrestorative sleep (0-No; 1-Sometimes; 2-Frequently; 3-Always)<br>Asphyxia crises (0-No; 1-Sometimes; 2-Frequently; 3-Always)<br>Nocturia (0-No; 1-Sometimes; 2-Frequently; 3-Always)<br>Witnessed apneas (0-No; 1-Sometimes)<br>Headache (0-No; 1-Sometimes)<br>Insomnia (0-No; 1-Starting; 2-Persistent)<br>Daytime sleepiness (0-No; 1-Sometimes; 2-Frequently; 3-Always)<br>Night awakenings (0-No; 1-Sometimes; 2-Frequently; 3-Always)<br>Cataplexy (0-No; 1-Yes)<br>Real or vivid dreams (0-No; 1-Yes)<br>Somnambulism (0-No; 1-Yes)<br>Sleep interrupted by heartburn (0-No; 1-Yes)<br>Grind teeth (0-No; 1-Yes)<br>Sleep duration (Holidays) (h)<br>Nap (0-No; 1-Yes)
Anthropometric	Weight (kg), Height (cm), Neck (cm), Waist (cm), Hips (cm), Body Mass Index (BMI)
Blood Test	Glucose, Urea, Creatinine, Cholesterol, High-Density Lipoprotein, Low-Density Lipoprotein, Triglycerides, Insulin, C-reactive protein, Thyroid-stimulating hormone, Thyroxine, Leukocytes, % Neutrophils, % Lymphocytes, Red blood cells, Hemoglobin, Platelets, Apolipoprotein E (APO-E)
Others	Decreased sexual desire or potency (0-No; 1-Yes)<br>Systolic blood pressure<br>Diastolic blood pressure
